# Transcriptomic Remodeling of Pulmonary Vein Sleeves Suggests a Role in Atrial Arrhythmogenesis in Thoroughbred Horses

**DOI:** 10.1111/nyas.70170

**Published:** 2025-12-15

**Authors:** Magdalena Arevalo‐Turrubiarte, Charlotte E. Edling, Carla Moller‐Levet, Bronte Forbes, Victoria Kemp, Joe Weir, Celia Marr, Rebecca Lewis, Kamalan Jeevaratnam

**Affiliations:** ^1^ School of Veterinary Medicine, Faculty of Health and Medical Sciences University of Surrey Surrey UK; ^2^ School of Biosciences, Faculty of Health and Medical Sciences University of Surrey Surrey UK; ^3^ Hong Kong Jockey Club, Sha Tin racecourse Sha Tin Hong Kong; ^4^ Rossdales Equine Hospital and Diagnostic Centre Newmarket UK

**Keywords:** atrial fibrillation, cardiac ion channels, cell types, equines, *Equus caballus*, myocardial sleeves, pulmonary veins, RNA sequencing

## Abstract

The initiation and maintenance of atrial fibrillation have been associated with physiological alterations in myocardial sleeves of the pulmonary veins (PVs). Gene expression profiles of the myocardial sleeves of the PVs in healthy (*n* = 3) and paroxysmal atrial fibrillation (PAF) (*n* = 6) thoroughbred horses (*Equus caballus*) were studied. Tissue collected from the left superior PV, adjacent to the left atrium, was analyzed by RNA sequencing. Gene Set Enrichment Analysis demonstrated positive enrichment of gene ontology biological processes related to muscle and endothelial cell development, cell shape, and structure organization in horses with PAF. Enrichment analysis of cellular and molecular functions showed upregulation of genes involved in transport and extracellular matrix components in horses with PAF. *SCN5A* and *MYH7*, which are associated with cardiac electrophysiology and contraction function, were both significantly upregulated in the PAF group (fold change 1.1 and 2.2, respectively). Cell deconvolution revealed a trend, although not significant, of increased numbers of fibroblasts. Our results suggest there are transcriptomic alterations in myocardial sleeves of PVs in horses with PAF, alterations related to both cardiac electrophysiology and tissue structure. These data shed further light on the potential role of PV myocardial sleeves in the pathophysiology of PAF.

## Graphical abstract

Myocardial sleeves of the pulmonary veins in racehorses with paroxysmal atrial fibrillation exhibit upregulated pathways associated with tissue structure remodeling. Pathways related to the extracellular matrix and cellular components differ significantly from those in healthy horses. A trend toward increased fibroblast expression in affected horses suggests that fibroblast activation may contribute to the progression of the condition.

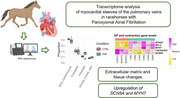

## 1 Introduction

Atrial fibrillation (AF) is a common cardiac dysrhythmia characterized by an uncoordinated and rapid atrial heart rate. The structure and function of the atria during AF are affected by the abnormal propagation of excitatory wavefronts [1, 2]. Studies of the underlying mechanism of AF in human patients have revealed that ectopic beats from the pulmonary veins (PVs) might be involved in the initiation and perpetuation of AF [3]. The electrophysiological connection between the PVs and the atria was suggested more than 40 years ago when a study in rats showed that sinus node‐like cells and membrane‐bound granules in the myocardial tissue of the pulmonary venous wall had structural similarities with those found in the atria [4]. In the left atrium, myocardial sleeves are described as nonuniform structures, with muscle fibers intersecting each other and extending into the PVs, exhibiting a larger length in the superior compared to the inferior PV region. In human hearts, this particular fiber arrangement indicates susceptibility to electrical instability in the atria [5]. Chen et al. [6] demonstrated that stimulation from chronic rapid atrial pacing in dogs affects PV rhythm, shortening its action potential duration compared to controls. Another study using electrocardiogram analysis and catheter mapping in human patients with AF revealed a re‐entrant circuit at the PV‐left atrial junction, with local stimuli originating from the distal PV [7]. Moreover, an in vitro study on canine cardiomyocytes from the PVs demonstrated greater slow and rapid rectifier currents and smaller L‐type calcium currents compared to those in the atria, indicating the PV's predisposition to abnormal rhythms [8].

Cardiac ion channels are membrane‐bound proteins found in cardiomyocytes that regulate conduction and electrophysiological activity. Research on proteins associated with cardiac ion channels in dogs shows differences in their expression between atria and PVs. For instance, the Na^+^/Ca^2+^ exchanger, the voltage‐gated potassium channel K_v_7.1, and ether‐a‐go‐go have been shown to exhibit higher expression in the cardiomyocytes of the PVs compared to the left atrium [9]. Moreover, it has been found that the heterogeneity in the expression profiles of ryanodine receptor 2 (RYR2), and the voltage‐dependent, L‐type and alpha 1C subunit (Ca_v_1.2) in the study of cardiomyocytes from rat PVs, together with the diverse organization of tubular networks, contribute to dysrhythmia [10]. This may reflect the higher frequency of spontaneous calcium release (known as calcium sparks) in the PVs compared to the left atrium and ventricle [10]. The evaluation of cardiac ion channels and other genes involved in the electrophysiology of the heart could help elucidate their potential as therapeutic targets.

In racing horses, AF causes suboptimal performance, with sudden deceleration and exercise intolerance. Paroxysmal atrial fibrillation (PAF) is defined as AF that reverts to sinus rhythm within 7 days. AF is commonly diagnosed in racing horses, and it has been suggested that horses could be used as a model to understand this pathology in humans [11]. The influence of PVs in horses with AF has not been extensively investigated compared to other species and remains incompletely understood. Spontaneous PV triggers in the equine heart have been demonstrated by Linz et al. [12] in a case report study. They observed that the conduction velocity of the PVs was faster compared to the left atrium. Furthermore, another study in horses showed that the myocardial sleeves of the PVs exhibit heterogeneous conduction velocity and higher collagen deposition compared to the left atria [13].

Research on the involvement of myocardial sleeves of the PVs in the initiation and maintenance of AF may aid in understanding this pathology in racing horses and human athletes. Recently, Edling et al. [14] demonstrated that in the healthy racing horse, genes involved in calcium regulation are more highly expressed in the atria compared to the ventricle. This finding highlights that region‐specific regulation within the heart contributes to cardiac electrophysiology and may influence disease susceptibility. The aim of the present study was to evaluate gene expression, with a focus on cardiac ion channels and genes associated with myocardial structure and fibrotic remodeling in the myocardial sleeves of PVs (principally, the left superior PV, adjacent to the left atrium) in healthy racing horses compared to those with PAF.

## 2 Materials and Methods

### 2.1 Animals and Tissue Collection

Ten thoroughbred racing geldings (*Equus caballus*), from 5 to 8 years old, were selected for our study (retrospective convenience sampling, clinical data are summarized in Table S1). They were clinically evaluated through cardiac auscultation and electrocardiogram analysis. The horses were classified into two groups: those with a history of PAF (*n* = 7) (horses with at least one episode of AF lasting up to 120 h) and control horses (*n* = 3) (healthy horses were defined as horses with no history of clinical cardiac abnormalities). The horses were euthanized due to musculoskeletal reasons over the course of a year, and a general necropsy was performed on the thorax to detect abnormalities—in accordance with the local authorities’ regulations of the Hong Kong Jockey Club. The left superior PV was targeted for sampling adjacent to the left atrium. This location was chosen as this tissue was more likely to be preserved intact and readily identifiable after post‐mortem retrieval of the heart. Equines have approximately four PVs [13, 15]; however, for the purposes of our study, we refer to the sampling location, which was the region we examined, as myocardial sleeves of the PVs. Biopsy samples were collected from the hearts using a disposable 5.0 mm skin biopsy punch (#20ID D52451, Kai Medical). Approximately 0.5−1 cm^3^ of tissue was obtained from and preserved in RNAlater (#R0901, Sigma).

### 2.2 RNA Extraction, Quantification, and Quality Assessment

Tissue (∼50 mg) was weighed using an analytical balance, ensuring clean conditions with RNAseZap solution (#R2020, Sigma). The tissue was homogenized with scissors and a Stuart homogenizer SHM1, in 600 µL of RNA Lysis buffer. RNA was extracted following the Monarch Total RNA Miniprep Protocol (#T2010S, New England Biolabs). The optional On‐column DNAse I treatment for enzymatic removal of residual genomic DNA step was performed, and total RNA was recovered in 50 µL of RNase‐free water, then preserved on ice for further analysis. Total RNA yield was measured with a spectrophotometer (CLARIOStar, BMG Labtech), and sample purity was assessed by the 260/230 ratio. RNA integrity was evaluated using the RNA 6000 RNA Nano Kit (#5067‐1511, Agilent) according to the manufacturer's instructions on a 2100 Bioanalyzer (Agilent). Samples for RNA sequencing analysis had RIN values ranging from 5.6 to 8. Total RNA samples were stored at −80°C prior to analysis.

### 2.3 RNA Sequencing

RNA‐seq library preparation, sequencing, and mapping were performed by Novogene according to their standard protocols. Briefly, messenger RNA was purified from total RNA using poly‐T oligo‐attached magnetic beads and processed for nondirectional library construction. Sequencing using Illumina Sequencing PE150 yielded clean read rates of 97.9–98.6% for all samples except HK08PV (94.3%, due to adapter contamination). Clean reads were aligned to the *Equus caballus* reference genome (Ensembl EquCab3.0, GCA_002863925.1) using HISAT2, with per‐sample error rates ≤0.03. Each sample generated 44.1–55.7 million reads, with 97.2–97.5% mapping rates (HK11: 96.2%, HK08: 94.4%). Exonic reads accounted for 66.8–71.0% in most samples, with lower values for HK11 (62.1%) and HK08 (55.4%). Notably, even though the RNA RIN values were on the modest side, the unique mapping rate was high and even across the samples (87–90%).

### 2.4 Data Analysis and Statistics

Gene count data was obtained, and the bioinformatic analysis was performed using R version 4.3.3. The bioinformatics workflow is visualized with details in Figure S1. The raw data consisted of 34,328 genes. Genes with “novel” gene annotation were excluded from the analysis, resulting in a total of 31,217 known genes. Further filtering was performed to remove lowly expressed genes (genes with at least 20 counts in at least three samples were retained) and noncoding biotypes, retaining only protein‐coding genes with annotations (12,513 genes).

To assess sample similarity, a regularized log transformation was applied using the *rlog* function from the Deseq2 package. Outliers were defined as those whose pairwise correlation average exceeded 1.5 times the interquartile range using Pearson correlation. One sample from the PAF group (HK_36) was identified as an outlier and excluded from the analysis, leaving three controls and six PAF samples for the downstream analysis (pairwise correlation and principal component analysis [PCA] plots are available in Figure S2). After the removal of the outlier sample, the filtering steps were redone, resulting in a total of 10,870 genes.

Because we observed nonexplained variability across samples and a lack of group clustering, we estimated the presence of hidden sources of variation in the data with the RUVseq package [16]. The RUVg option of RUVseq estimates latent factors based on empirically defined control genes (5000 genes with the least variation [FC near 0]) from a Deseq2 differential expression (DE) analysis with no covariates in the design. Then, the factors were used as covariates in the Deseq2 DE analysis to remove the unwanted variation (batch correction). To control for multiple testing, a false discovery rate was applied to the *p*‐values using the Benjamini−Hochberg method. A table with the Deseq2 DE analysis statistics for all genes is available in Table S2. The batch corrected expression values were used for the plotting of the PCA using the *prcomp* function and *ggplot2* package. PCA plots, *p*‐value histograms, MA plots, heatmaps, and Venn diagrams showing the effect of the batch correction are available in Figures S3–S6.

Gene Set Enrichment Analysis (GSEA) was performed with Gene Ontology (GO) biological processes (GO_BP), molecular functions (GO_MF), and cellular components (GO_CC) databases, with the species set for *Equus caballus* retrieved with the package *msigdbr*. Genes were ranked by fold change, and the enrichment was analyzed using the package *fgsea*. Gene sets of sizes 20–400 genes were included, and 10,000 permutations were done within the *fgsea* function. After removing redundant terms (collapsing), the main terms with adjusted *p*‐value <0.05 were ranked by absolute normalized enrichment score (NES), and the top 20 terms were displayed.

Heatmaps were generated with the package *pheatmap* using RUVg batch corrected and normalized, log2 transformed expression values, scaled by row for color coding, but with no clustering. Selection of genes to represent electrophysiology and contraction‐relevant genes were based on our previous work in horses [14, 17]. Genes selected as fibroblast/extracellular matrix (ECM) markers were based on publications from horses [18], humans [19], mice [20], and the Human Protein Atlas (www.proteinatlas.org, search: ce_enriched:heart muscle;fibroblasts;Very high,High,Moderate). The use of human cardiac data as reference is supported by the evident similarities between human and horse heart structure, function, and potential for spontaneous AF [21], and a quantitative proteomics study comparing cardiac tissue across species, which demonstrated that, based on ∼7000 proteins, the human and horse heart proteomes are overall similar to each other [22].

BayesPrism cell deconvolution was used to investigate the tissue cell composition following the published method [23] and [24]. The gene signatures for each cell type were based on the top 90–200 most significant marker genes extracted from single‐cell RNA sequencing data from human right atria using the *BayesPrism* package [24]. Identified cell types included adipocytes, fibroblasts, endothelial, lymphoid, myeloid, neuronal, pericytes, smooth muscle cells, and atrial cardiomyocytes. The equine gene ensemble names were matched to human gene symbols using BiomaRt [25]. The cell proportions obtained from the analysis were plotted as standard boxplots and evaluated statistically using the Wilcoxon test with the Benjamini−Hochberg correction applied to the *p*‐values. Human RA signature data was used since horse‐specific single‐cell RNA sequencing data is not available. As discussed previously in this paper, horses and humans have very similar heart structure and function, and hence, it is possible to use human cardiac RNA data as a reference.

The volcano plot was produced using *ggplot2*. The top 20 DE upregulated and downregulated genes, with a log2 fold change greater than 1, a baseMean>50, and an adjusted *p*‐value < 0.05, were labeled in the plot.

## 3 Results

### 3.1 Upregulated Genes Associated With PAF Were Enriched for Muscle Structural and Cytoskeletal Organization Processes

**FIGURE 1:**
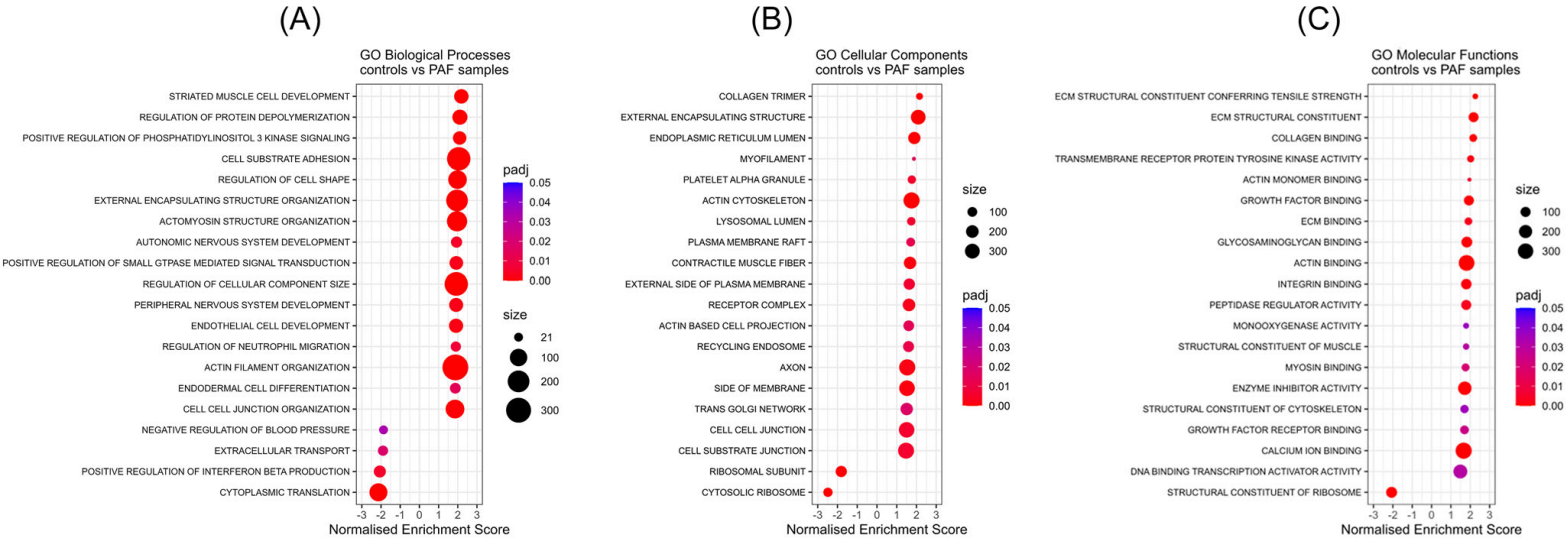
Gene set enrichment analysis of control versus PAF in myocardial sleeves of the PVs. The analysis was performed using gene ontology (GO) databases with the complete list of genes ranked by fold change. (A) Shows the GO biological processes, (B) the cellular components, and (C) the molecular functions. Plots display the 20 top main terms with adjusted *p*‐value <0.05, ranked by absolute normalized enrichment score (NES). Dot colors indicate significance, and the size indicates the number of genes included in the term. Positive NES indicates activation, and negative indicates suppression of terms in PAF horses. Abbreviation: PAF, paroxysmal atrial fibrillation.

The DE genes were ranked by fold change and mapped to GO biological processes, molecular functions, and cellular components. After collapsing to main pathways, 86 terms had an adjusted *p*‐value<0.05 for the biological processes, 26 terms for the cellular functions, and 20 for the molecular functions. The top 20 terms ranked by largest absolute NES are shown in Figures 1A−C. Contrasting control against PAF showed positive enrichment of GO biological processes terms, including “striated muscle cell development,” “cell substrate adhesion,” “regulation of cellular component size,” and “actin filament organization.” On the other hand, genes were expressed at lower levels in terms related to “extracellular transport,” “cytoplasmic translation,” and “negative regulation of blood pressure” (Figure 1A). Similarly, GO cellular components showed positive enrichment of terms such as “collagen trimer,” “external encapsulating structure,” “contractile muscle fiber,” “actin cytoskeleton,” and “axon” in the PAF horses, while terms related to the ribosome were downregulated (Figure 1B). GO molecular function analysis highlighted high expression of genes associated with the ECM, including terms such as “ECM structural constituent,” “collagen binding,” “actin binding,” and “calcium binding.” In contrast, the term “structural constituent of ribosome” showed reduced gene expression in this analysis as well (Figure 1C).

### 3.2 Electrophysiology and Contraction‐Related Genes *CACNA2D3*, *KCNN2*, *SCN5A*, *MYH7*, and *MYL2* Expression Levels Differ Significantly Between Control and PAF Groups

**FIGURE 2:**
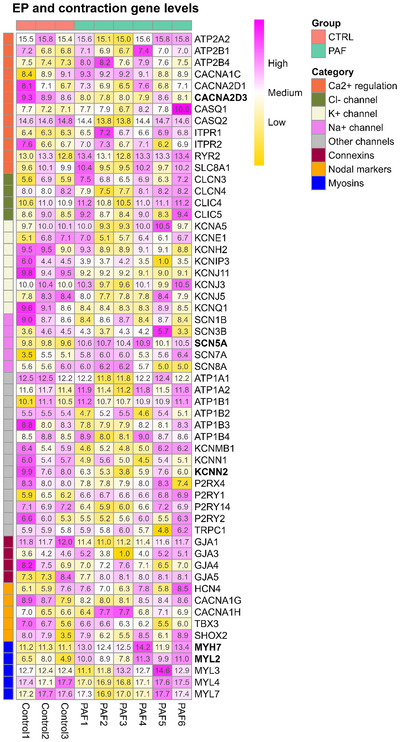
Gene expression levels of selected cardiac ion channels and electrophysiology‐related genes in the myocardial sleeves of the pulmonary vein in controls and PAF. Heatmap with genes coding for cardiac ion channels and electrophysiology‐related proteins. The genes are grouped by ion preference or function into eight categories: Ca^2+^ regulation, Cl^−^ channels, K^+^ channels, NA^+^ channels, other channels, connexins, nodal markers, and myosins. Bold gene names indicate significantly differentially expressed genes (adjusted *p*‐value<0.05). Values in the heatmap are log2 transformed counts, normalized and corrected for batch effect. Pink represents higher, and yellow represents lower, expression levels. Abbreviation: PAF, paroxysmal atrial fibrillation.

We next investigated the main cardiac ion channels and electrophysiology‐related genes described in horses [14]. The selected genes were visualized using a heatmap to evaluate their expression patterns in both groups (Figure 2).

Among genes involved in calcium regulation, *ATP2A2* (SERCA2), *CASQ2* (calsequestrin 2), and *RYR2* (ryanodine receptor 2) were the most abundantly expressed in both groups, consistent with their central role in regulating calcium flux via the sarcoplasmic reticulum (SR). No marked differences in expression were observed between PAF and control samples for these genes. In contrast, *CACNA2D3*, encoding the calcium voltage‐gated channel auxiliary subunit α2δ3, was significantly downregulated in PAF (adjusted *p*‐value = 0.019; log_2_FC = –0.76).

Potassium ion channels regulate and maintain the stability of action potentials. They also contribute to the repolarization reserve. Our analysis showed that *KCNA5*, which encodes K_v_1.5, a channel involved in the regulation of atrial action potential repolarization in the heart, was highly expressed in both controls and PAF horses. Also, *KCNJ3*, which encodes the potassium inwardly rectifying channel subfamily J member 3 (Kir3.1), was highly expressed in both groups. Notably, the small conductance calcium‐activated potassium channel protein 2 (SK2), encoded by *KCNN2*, showed significantly lower expression (log2 FC = −2.53, adjusted *p*‐value = 0.035) in PAF compared to controls (Figure 2).

Consistent with our previous findings in the horse heart [14], sodium channel, *SCN5A*, encoding for Na_v_1.5, was highly abundant compared to other genes encoding sodium channels. Interestingly, SCN5A, which is responsible for the fast‐inward sodium current that initiates the action potential upstroke in cardiomyocytes, was significantly upregulated in PAF compared to controls (log2 FC = 1.11, adjusted *p*‐value = 0.00012).

When exploring myosin expression in the myocardial sleeves of the PV, we found that MYL4 and MYL7, which encode myosin light chains 4 and 7, respectively, showed higher expression levels than other myosins in our analysis. Distinctively, MYH7, encoding the beta myosin heavy chain subunit, and *MYL2*, which encodes myosin light chain 2, were significantly upregulated (*MYH7* with log2FC = 2.23 and adjusted *p*‐value = 0.000011; *MYL2* with log2FC = 3.73 and adjusted *p*‐value = 0.0045) in PAF compared to controls (Figure 2).

Genes associated with pacemaker activity and nodal cell phenotype (*HCN4*, *CACNA1G*, *CACNA1H*, *TBX3*, and *SHOX2*) were included due to their potential role in PV ectopy. However, none of these genes showed significant differences in expression between PAF and control groups.

Overall, while a subset of electrophysiology‐ and contraction‐related genes, notably *SCN5A*, showed significant differential expression between PAF and control groups, the majority of genes examined did not display group‐dependent expression patterns in our data set, suggesting that the core transcriptional landscape of ion handling in PV myocardial sleeves was largely preserved in PAF (Figure 2).

### 3.3 A Nonsignificant Increase in Fibroblast Proportion Was Observed in PAF Compared to Controls

**FIGURE 3:**
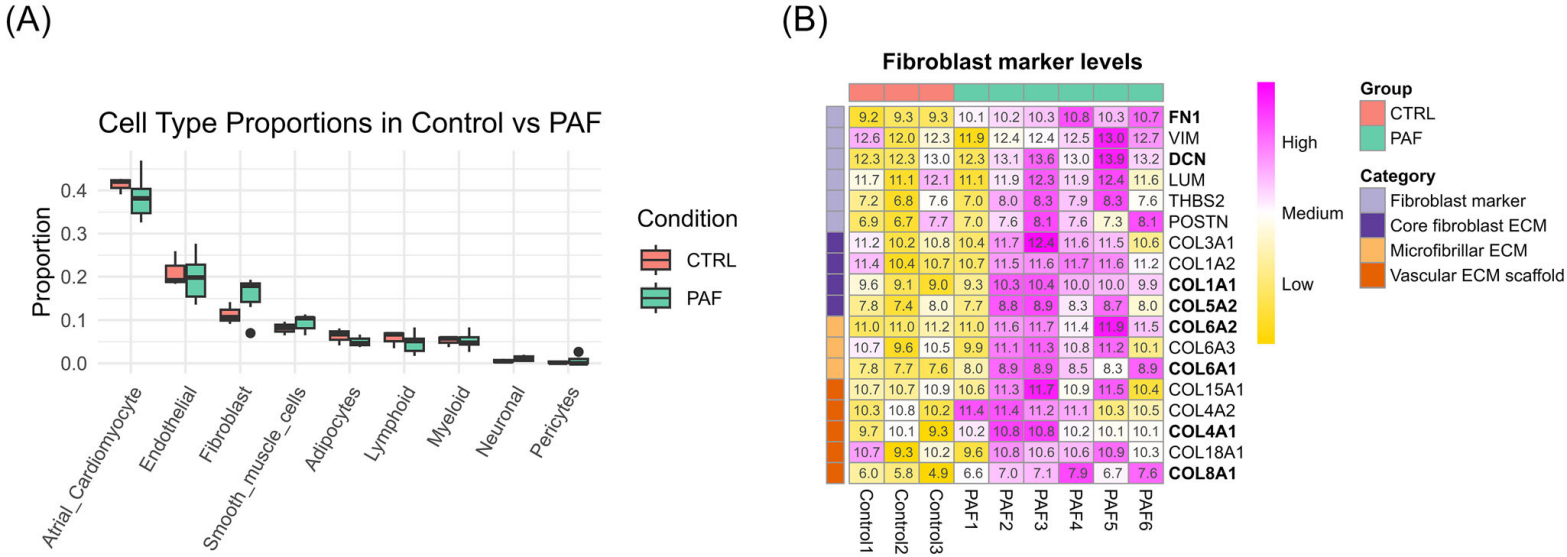
Cell deconvolution and fibroblast marker analysis of myocardial sleeves of the pulmonary veins in healthy and PAF horses. (A) Cell deconvolution of the RNA sequencing data from control and PAF samples was performed using BayesPrism with signature marker gene profiles from human atria. Percentage data from each group and cell type was plotted as standard boxplots, with percentages indicating the proportion of each cell type in control (red) and PAF (green) samples. Data was statistically tested by Wilcoxon test. No significant differences between control and PAF were observed in any cell type. (B) Heatmap of genes coding for collagens and other fibroblast markers. The genes are grouped into four categories by type: fibroblast markers, core fibroblast ECM, microfibrillar ECM, and vascular ECM scaffold. Values in the heatmap are log2 transformed counts, normalized and corrected for batch effect. Bold gene names indicate significantly differentially expressed genes (adjusted *p*‐value<0.05). Pink represents higher, and yellow represents lower, expression levels. Abbreviations: CTRL, control; ECM, extracellular matrix; PAF, paroxysmal atrial fibrillation.

Cell deconvolution analysis was performed to estimate cell proportions within the myocardial sleeves of the PVs. To identify the cell types, we employed a cardiac signature derived from the human right atrium. Analysis of gene expression profiles revealed the presence of atrial cardiomyocytes, endothelial, fibroblasts, smooth muscle cells, myeloid, adipocytes, lymphoid, neuronal, and pericytes (Figure 3A). Overall, atrial cardiomyocytes were the most abundant cell type in both groups, with a slightly higher proportion in the controls compared to the PAF group. Notably, the PAF group exhibited a higher, however, nonsignificant, proportion of fibroblasts compared to the control group (Control‐mean = 11%, PAF mean 16%, Wilcoxon Benjamini−Hochberg adjusted *p*‐value = 0.68). To further explore the possibility of increased fibroblast presence in the PAF horses, we extracted known conserved fibroblast marker genes and collagen genes from the expression data and plotted them as a heatmap (Figure 3B). Overall, the heatmap shows a clear pattern of increased expression in most of the PAF samples compared to the control samples. Two of the general fibroblast markers were significantly more expressed in PAF; *FN* coding for fibronectin and *DCN* coding for decorin (*FN* with log2FC = 1.44 and adj.p = 1.1E‐11, *DCN* with log2FC = 0.95 adj.p = 0.038), indicating greater fibroblast activity and ECM remodeling. In addition, several of the collagens were significantly upregulated, suggesting increased core ECM, microfibrillar ECM, and vascular ECM scaffold (Figure 3B).

### 3.4 The Most Significantly Expressed Genes Represent a Mix of ECM Remodeling, Contractile Proteins, Ion Channels, Regulatory Factors, and Stress Response

**FIGURE 4:**
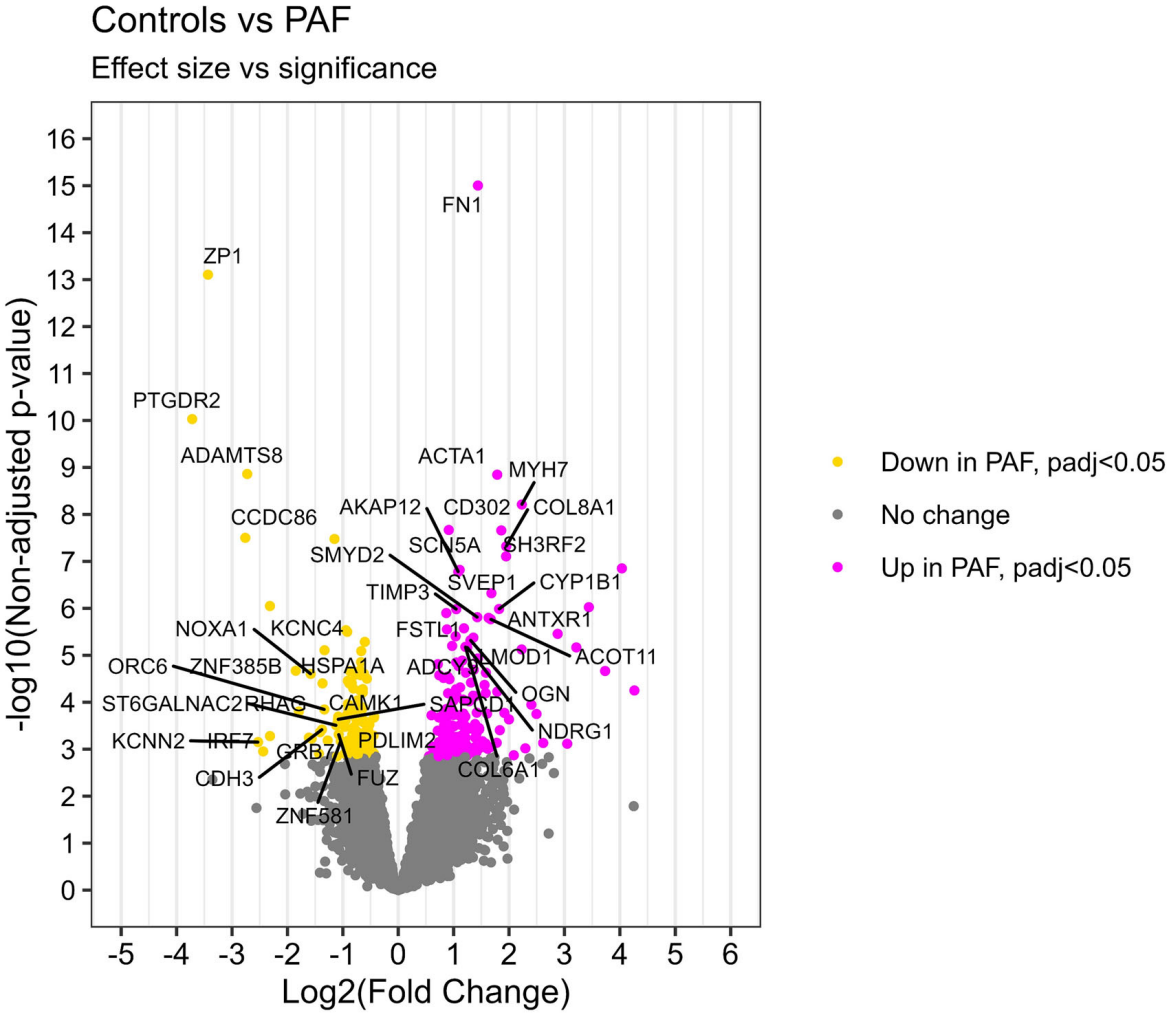
Volcano plot of all expressed genes from myocardial sleeves of the pulmonary veins in control versus PAF. One hundred and sixty‐eight genes, colored pink, were significantly (p‐adj <0.05) upregulated in PAF, 135 genes, colored yellow, were significantly (p‐adj <0.05) downregulated in PAF samples. The top 20 upregulated and the top 20 downregulated genes, with an absolute log2 fold change >1 and a basemean>50, were labeled with gene name. Abbreviation: PAF, paroxysmal atrial fibrillation.

The volcano plot (Figure 4) visualizes the most significantly regulated genes at an individual level. Genes labeled in the plot are the top 20 most significant, by *p*‐value, down‐, respectively, up‐regulated genes with an absolute fold change larger than 1 and expression level higher than BaseMean 50. This again demonstrates strong upregulation of ECM and fibroblast‐associated genes, including *FN1*, *COL6A1*, *COL8A1*, *SVEP1*, *TIMP3*, and *OGN*, consistent with enhanced matrix remodeling. Contractile and cytoskeletal components (*ACTA1*, *MYH7*, *LMOD1*) were also upregulated, while the structural adaptor *PDLIM2* was reduced. As discussed previously, some ion channels were significantly differentially expressed; *SCN5A* and *KCNN2* are labeled in the volcano plot because of their large fold change. Transcriptional regulators were widely affected, with increases in *SMYD2*, *AKAP12*, and *NDRG1*, while factors such as *IRF7*, *GRB7*, and *ZNF385B* were downregulated. Additionally, *HSPA1A* (encoding the heat shock protein Hsp70‐1A) and *NOXA1* (a regulator of NADPH oxidase–mediated ROS production) were both downregulated, indicating a potential weakening of protective mechanisms against protein misfolding and oxidative stress (Figure 4).

## 4 Discussion

In horses, the existing literature on the myocardial sleeves of the PVs in the context of AF is limited. Linz et al. [12] demonstrated spontaneous electrical activity in the left superior PV during catheter placement. Kjeldsen et al. [13] reported higher conduction activity in the left PVs and significantly higher collagen deposition in the myocardial sleeves of the PVs compared to the atria. However, the role of the PVs in the initiation and maintenance of AF in the horse has not been investigated as much as it has been in humans. In this study, we evaluated gene expression profiles and tissue changes in the myocardial sleeves of the PV in healthy and PAF racing horses with the aim to contribute to the understanding of the role of the PVs in this pathology.

The pathway analysis of differentially expressed genes in the myocardial sleeves of the PVs revealed a significant upregulation of genes associated with muscle organ development in horses with PAF. Structural changes in cardiac tissue, such as atrial enlargement, have been previously reported in the left atrium of horses with chronic AF [2]. Although measurements of the left atria size were not available in our study, the increased expression of genes related to muscle organ development may reflect ongoing structural adaptations in the cardiac tissue. This is consistent with findings from a study that demonstrated a decline in atrial function within just 3 days of AF induction in horses [26]. In our cohort study, episodes of heart rhythm irregularities (PAF) were detected several months prior to euthanasia of the horses, suggesting that structural alterations may have already been in progress in the PAF group.

Our results further support the presence of tissue structural changes in the myocardial sleeves of the PVs in PAF by the significant increase in terms involved in the actin filament organization and the regulation of cellular component size. Previous studies in rats have shown that altered cardiac load can lead to changes in the myocyte size and increased frequency of calcium release [27]. In our samples, expression of *RYR2* and *SLC8A1* was unchanged, while *CACNA2D3* was significantly reduced, suggesting that L‐type calcium channel–mediated calcium entry may be altered during PAF, potentially contributing to electrophysiological remodeling.

The ectopy of the PVs as a source has been described in humans by Haïssaguerre et al. [3]. We, therefore, analyzed genes involved in cardiac ectopy, such as *HCN4*, mutations of which can disrupt normal pacemaker function. However, differences in genes relevant to pulmonary ectopy were not significant between controls and horses with PAF in our study. This may be due to the relatively young age of the horses, in contrast to dogs, where studies have shown significant expression in older individuals with AF [28], which often represents a more persistent arrhythmia rather than PAF episodes. In addition to the PVs, ectopic activity arising from the vena cava has also been implicated as a potential trigger of AF in horses [29]. Further research is warranted to determine whether these venous triggers share common molecular mechanisms or reflect distinct pathways contributing to the initiation of PAF.

When we examined cell type proportions in the myocardial sleeves of the PVs, we observed a trend toward a reduction of atrial cardiomyocytes and an increased presence of fibroblasts in PAF. In the healthy heart, fibroblasts are known to maintain the cardiac tissue structure. However, in response to injury, they contribute to tissue repair by promoting scar formation and wound healing. Our results indicate that ECM alterations might be taking place as the PAF pathology progresses. An in vitro study using cells from human patients with AF demonstrated increased differentiation of fibroblasts into myofibroblasts, indicating a phenotypic change associated with the arrhythmogenic environment [30]. Although no significant differences in the cell type proportions were observed in our study, the indicated shifts in cell proportions between the two groups, supported by significant upregulation of fibroblast marker genes, may reflect tissue adaptations to arrhythmias in PAF horses. A limitation of our analysis is that the cell type deconvolution relied on human atrial single‐cell data as a reference, since horse single‐cell data are not yet available; therefore, species‐specific differences in transcriptional profiles may influence the resolution of fibroblast abundance estimates.

Fibroblast cell differentiation has been associated with an increase in sodium currents, particularly Na_v_1.5, suggesting that the emergence of *de novo* currents may result from changes in cell phenotype and regulatory mechanisms [31]. Our findings support the notion of increased ion currents such as Na_v_1.5. However, our study investigated these ion channels through their gene expression patterns rather than electrophysiological methods. *SCN5A*, which encodes the sodium channel Na_v_1.5, was increased in the myocardial sleeves of the PVs in horses with PAF, suggesting an enhanced depolarization phase of the electrical action potential. Dysfunction of Na_v_1.5 is commonly associated with *SCN5A* mutations, where its gain‐of‐function contributes to AF [31]. In addition to the effects of *SCN5A* mutations on sodium handling, it is known that Na_v_1.5 interacts with other molecules from the cytoskeleton and ECM, and gain‐of‐function mutations in *SCN5A* have been shown in a mouse model to alter cardiac fibroblasts, leading to increased proliferation and fibrosis [32]. Hence, the changes in fibroblast proportions observed in our study might be linked to the increased SCN5A levels, which together imply remodeling as is usually observed in the left atria during AF.

Overall, our results provide an overview of cardiac electrophysiology‐relevant genes present in the myocardial sleeves of the PVs that could be further investigated as potential therapeutic targets in AF associated with PVs.

Even though Na_v_1.5 may potentially be important in the treatment context, its use as a direct therapeutic target is complicated by its cardiac wide expression pattern; however, its broader impact on the regulation of other ion channels also warrants further investigation. We observed a similar downregulation of the *KCNN2* gene in the myocardial sleeves of the PVs as previously reported in atrial tissue from human patients with PAF [33]. In contrast, in human AF, there is an enhanced sensitivity of the SK2 currents to calcium, which contributes to a reduction in action potential duration [34]. *KCNN2* encodes the SK2 channel, which plays a role in the repolarization process. Reduced expression of *KCNN2* may be associated with dysregulation of the action potential in the myocardial sleeves of the PVs. When exploring molecular functions through GO analysis, positive enrichment was identified in ECM, collagen binding, and actin binding terms. An increased percentage of the ECM area fraction has previously been observed in dogs with sustained AF [35]. Particularly, upregulation of several of the most abundant collagen genes *COL1A2*, *COL3A1*, and *COL6A2* were found in our study, demonstrating changes in the components of the ECM. In contrast, *ADAMTS8*, encoding for a metalloprotease which is associated with the degradation of the ECM and in promoting cardiac fibrosis [31], was downregulated in our data. These gene expression changes may reflect remodeling and modulation within the myocardial sleeves of the PV during the progression of PAF. At the cellular level, the upregulation of the genes *MYH7*, *MYL2*, and *ACTA1*, linked to actin‐binding proteins and skeletal muscle, in our study suggests cytoskeletal alterations and changes in contraction within the PAF tissue. Cytoskeleton microfilaments are composed of actin‐binding proteins that regulate tissue dynamics, and these proteins have been associated with cardiac hypertrophy in humans [36].

Altogether, our results demonstrate changes in the myocardial sleeves of the PVs in PAF horses. This may suggest that remodeling could occur in these regions, potentially contributing to the increased susceptibility to electrical dysregulation observed in PAF horses.

While these findings provide new insights, the study is based on a limited number of opportunistically collected samples and is subject to natural biological variation among horses. Therefore, additional investigations of the myocardial sleeves of the PVs are warranted. In particular, studies incorporating orthogonal validation at the protein or histological level, and including chronic or permanent AF stages, would be valuable to elucidate the progression of this pathology in racing horses.

## 5 Conclusion

In conclusion, through gene expression analysis, this study demonstrates alterations in pathways related to the ECM, interactions between proteins and cell structural components and cytoskeleton, indicating structural and functional changes in the myocardial sleeves of the PVs in horses with PAF. In line with the structural remodeling, analysis of cell type proportions revealed trends in cell type shifts between control and PAF tissue. Additionally, differential expression of *SCN5A*, *KCNN2*, and *CACNA2D3* ion channel genes suggest a component of direct electrophysiological change in the progression of PAF in the myocardial sleeves of the PVs in horses. Given their involvement, these genes may serve as potential targets for further investigation. Collectively, our findings support the presence of cardiac tissue alterations in the myocardial sleeves of the PVs in PAF racing horses. This study contributes to the understanding of the role of the myocardial sleeves of the PVs during cardiac dysrhythmia in the early stages of the AF pathology progression in racing horses.

## Author Contributions

**M.A.‐T**.: Data curation, software, and writing. **C.E.E**.: Validation, software, writing and editing. **C.M‐L**.: Software, data curation. **B.F., V.K., J.W**.: Methodology and resources. **C.M**.: Conceptualization. **R.L**.: Validation, review, and editing. **K.J**.: Funding acquisition, conceptualization, and validation.

## Funding

This project was funded by the Hong Kong Jockey Club Equine Welfare Research Foundation grant number MGR‐2021‐101262.

## Conflicts of Interest

The authors declare no conflicts of interest.

## Ethical Approval Statement

Animals were euthanized according to the local authorities of the Hong Kong Jockey Club, Sha Tin racecourse, Sha Tin New Territories, Hong Kong. Followed by the Veterinarian officers, samples were collected under the rule 46 (clause 1 and 2). Ethical approval was given by the Self‐Assessment for Governance and Ethics‐Animal Research (SAGE‐AR) from the University of Surrey, United Kingdom, with an ID 638929‐638920‐76858336 number.

## Supporting information



Figure S1. Bioinformatics workflow with order of procedures and outcome of filtering steps.

Figure S2. Sample similarity and outlier detection.
(A) Sample similarity analysis by Pearson correlation. Samples with a mean correlation below 1.5 times the interquartile range below the first quartile were considered outliers and removed from further analysis. (B) PCA plot of rlog transformed filtered counts before and (C), after removal of outliers.

Figure S3. PCA plots to demonstrate effect of batch correction.
(A) PCA plot of log2 transformed refiltered (after outlier removal) normalized counts. (B) PCA plot of log2 transformed refiltered (after outlier removal) RUVg corrected normalized counts. (C) PCA of residuals from Deseq2 DE analysis without batch correction. (D) PCA of residuals from Deseq2 DE analysis with RUVg batch correction.

Figure S4. Control plots for DE analysis.
(A) *p*‐value distribution of *p*‐values from DE analysis of control versus PAF, uncorrected. (B) *p*‐value distribution of *p*‐values from DE analysis of control versus PAF with RUVg factor as covariant for batch correction. (C) MA plot (log fold change vs. log of normalized counts) from DE analysis without batch correction. (D) MA plot (log fold change vs. log of normalized counts) from DE analysis with RUVg factor as covariant for batch correction.

Figure S5. Venn diagrams for comparing effects of batch correction on DE analysis. (A) Venn diagram of genes with *p*‐value <0.05 from DE analysis with and without batch correction. 90% of genes with *p*‐value<0.05 from the DE analysis without batch correction overlap with the corrected genes with *p*‐value<0.05. (B) Venn diagram of genes with adjusted *p*‐value <0.05 from DE analysis with and without batch correction. All 17 significant genes without correction are also considered significant with batch correction.

Figure S6. Heatmap with clustering for comparing effects of batch correction on significant genes. (A) Heatmap of log2 uncorrected counts with padj<0.05 from the DE analysis. Without batch correction, 17 genes were considered significantly differentially expressed. The three control samples cluster together with one PAF sample on the right side. (B) Heatmap of log2 RUVg corrected counts with padj<0.05 from the DE analysis. With batch correction (removal of unwanted variation with RUVg), 303 genes were considered significantly differentially expressed. The three control samples cluster together on the right side, while the PAF samples cluster together on the left side of the heatmap.

Table S1. Characteristic of the horses in the study. Table shows information of the healthy (control) and PAF horses used in this study, along with a description of their main clinical findings before euthanasia. EIPH, exercise‐induced pulmonary hemorrhage; OA, osteoarthritis.

Table S2. Table of all genes analyzed for differential expression. Table with differential analysis statistics from the Deseq2 analysis.
