# Supplementary figures and images for: Transcriptomic Remodeling of Pulmonary Vein Sleeves Suggests a Role in Atrial Arrhythmogenesis in Thoroughbred Horses
Source: Ann N Y Acad Sci. 2025 Dec 15;1556(1):e70170. doi: 10.1111/nyas.70170 (PMC12917935; doi:10.1111/nyas.70170)

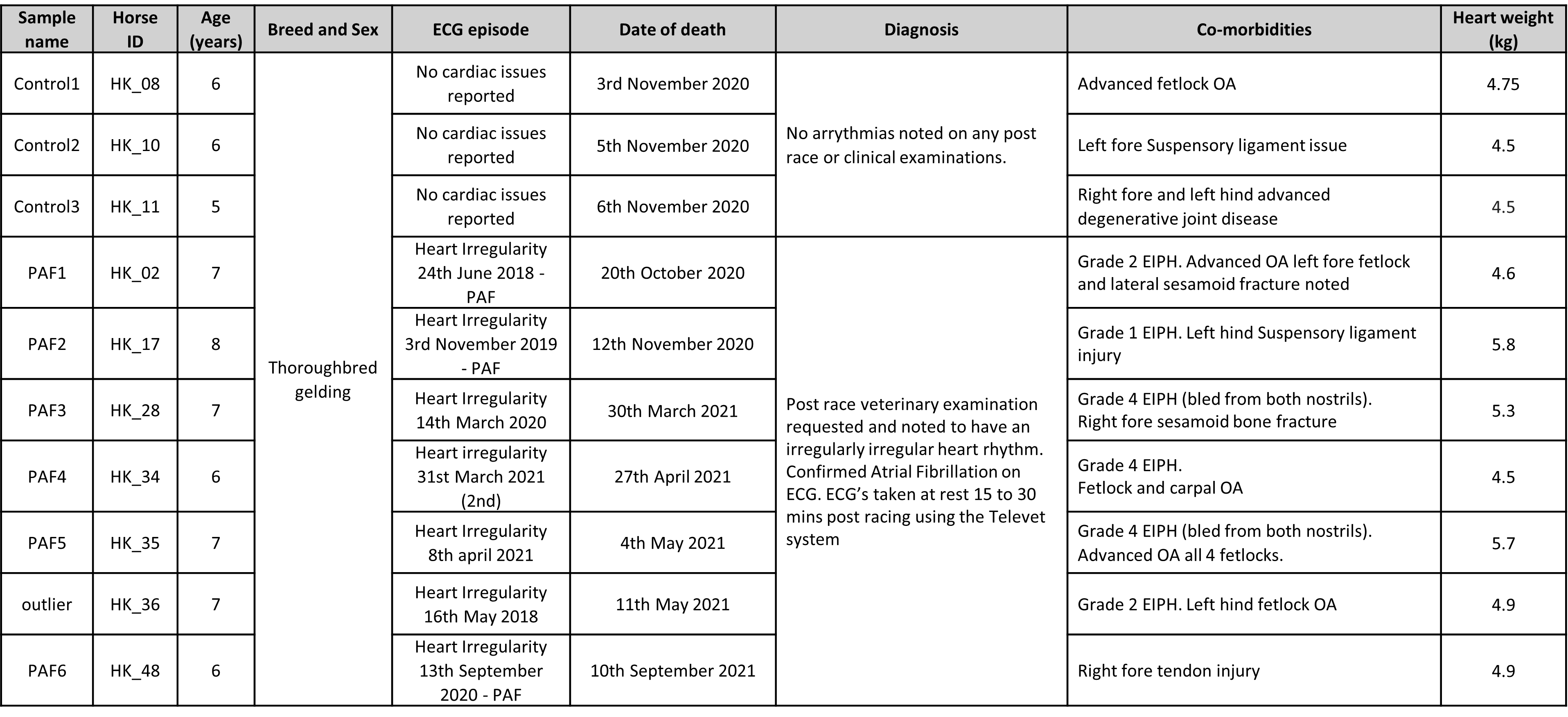

Supplement: Supplementary file 7 — Table S1. Characteristic of the horses in the study. Table shows information of the healthy (control) and PAF horses used in this study, along with a description of their main clinical findings before euthanasia. EIPH, exercise‐induced pulmonary hemorrhage; OA, osteoarthritis. [file NYAS-1556-0-s006.tif]
